# Kurt-Semm-Center of minimally invasive and robot-assisted surgery: Over 10 years of an interdisciplinary and interprofessional project in clinical application, research and training

**DOI:** 10.1007/s11701-026-03238-3

**Published:** 2026-02-23

**Authors:** Ibrahim Alkatout, Almut Kalz, Robert Bergholz, Kai Bachmann, Jan Henrik Beckmann, Jan-Hendrik Egberts, Joß Giese, Jonas Jarczyk, Grischa Hoffmann, Nicolai Maass, Philipp Nuhn, Daniar Osmonov, Göntje Peters, Julius Pochhammer, Benedikt Reichert, Severin Rodler, René Rusch, Terbish Taivankhuu, Thilo Wedel, Henning Wieker, Jörg Wiltfang, Thomas Becker

**Affiliations:** 1 University Hospital Schleswig-Holstein, Campus Kiel, Kiel, Germany; 2 Present Address: Israelitisches Krankenhaus, Hamburg, Germany; 3 Institute of Anatomy of Christian-Albrechts-University, Kiel, Germany

**Keywords:** Minimally invasive surgery, Robot-assisted surgery, Robotic surgery, Cross-disciplinary collaboration, Surgical training, RAS

## Abstract

We are looking back on over 10 years of successful work and numerous milestones in the interdisciplinary center of minimally invasive surgery, robotic surgery in particular. The success is largely owing to the spirit of sharing and collaborating — rather than competing — across the surgical disciplines. The collaboration became particularly fruitful by including the Kiel Center of Clinical Anatomy of Kiel University where many surgical approaches and techniques have been developed or refined on body donors. Apart from the traditional and renowned “Kiel school” for training in gynecological endoscopy built on the heritage of Kurt Semm; a training center (Kurt-Semm-Academy) for robot-assisted surgery was founded in 2020. The Kurt-Semm-Center has always followed the credo of establishing the best surgical technology for the best possible patient treatment. The ambitious claim to offer not only state-of-the-art surgery, but also to advance it further through studies and research, both experimental and clinical. This is evidenced by the enormous output of innovative research and publication activity, some of which is presented in this article. The key to success has been co-working across the disciplines, with continuing tenacity and dedication to state-of-the-art minimally invasive surgery. We believe that many hospitals could profit from an interdisciplinary spirit, having realized that individual department-specific interests are eventually better met when people learn from each other and work as a team. This article is dedicated to the spiritus rector of the Kurt-Semm-Zentrum, Prof. Dr. med. Klaus-Peter Jünemann.

## Introduction

The “Kurt-Semm-Zentrum” in Kiel was founded in 2015 as a collaborative network of the surgical disciplines at University Hospital of Schleswig–Holstein (UKSH), Campus Kiel, to promote the interdisciplinary clinical implementation and foster the further development of minimally invasive surgery, in particular of the then still young techniques and methods of robot-assisted surgery^1^.

We are looking back on 10 years of successful work and numerous milestones that have been largely owed to the spirit of sharing and collaborating—rather than competing—across the surgical disciplines. This retrospective article is dedicated to the spiritus rector of the Kurt-Semm-Zentrum, Prof. Dr. med. Klaus-Peter Jünemann^2^.

### Why Kurt Semm?

Kurt Semm^3^, a gynecologist and precision engineer of laparoscopic surgical instruments, was a pioneer of minimally invasive surgery in close cooperation with his brother and their joint instrument company, WISAP. In 1970 he became the director of the Gynecology Department at Kiel University Hospital and founded the Kiel School for Endoscopic Gynecological Surgery. He was the first to attempt laparoscopic surgery—removal of an appendix – in 1980. Not only was he dismissed from medical associations, he was also declared to be a lunatic and advised to seek psychiatric treatment. Obsessed with the unstoppable progress of laparoscopy, he eventually achieved fame and honor for his game-changing inventions.

### The story behind

Klaus-Peter Jünemann, Director of the Urology Department from 2001to2022) (deceased 2023), was one of the first in UKSH to read the sign of the times: that the future of surgery would be minimally invasive and that the new robotic surgery techniques, tremor-free, ergonomic, and with huge potential for increasing digital enhancements, would oust open surgery and eventually even laparoscopy. After many years of futile efforts, he succeeded in securing funds to implement the first two da Vinci Si-Systems at UKSH, Campus Kiel, in early 2013. He also realized, however, that collaboration rather than competition in using this new technology would promote broader application and accelerate development of minimally invasive surgical techniques. Thus, the idea was born to found the Kurt-Semm-Center (after official permission from the heirs of Kurt Semm to use the name had been obtained), which at the beginning was little more than a club of surgeons from urology, gynecology and general surgery and some other surgical disciplines, who met regularly to discuss how to tread new paths in surgery.

### The benefit of collaborating across disciplines

Co-working across disciplines in robot-assisted surgery has benefitted all involved and fostered collegiality and friendship among doctors from different departments. In the early years of robotic surgery, it was a habit from the start that each discipline introducing robotic surgery was supported by more experienced surgeons from other departments. Thus urologists, general surgeons and gynecologists would often work together in the OR, or support robotic surgeons from other disciplines in performing individual patient care. Apart from the steady expansion of the clinical spectrum and growth in case numbers, the goal has always been to take robotic surgery further. Collaboration with Kiel University’s Center of Clinical Anatomy (Institute of Anatomy) has enabled us to explore surgerical procedures, novel techniques and docking positions in body donors. Many interventions were thus developed or refined, such as esophageal surgery, bariatric and colorectal surgery, as well as special techniques in urological, gynecological and maxillofacial surgery. Furthermore, cross-disciplinary collaboration has boosted teamwork in robotic procedures, strengthened clinical and preclinical research, and stimulated research papers and projects. Despite the frequently criticized negative cost balance compared with conventional techniques, robotic surgery has been proven to have economically positive effects on surgical time, complication rates, hospitalization time, reoperation rates, etc. which may ultimately outweigh the higher immediate costs [1, 2].

One of the highlights underscoring the collaborative nature of the robotic surgery program was the hosting of the 2019 Meeting of the German Society of Robotic Urology (DGRU). The unique and visionary feature was to broaden the approach and perspective by including robotic general surgery and surgical gynecology as equal partners. There were multiple live transmissions from the OR, robotic surgeons of various disciplines from near and far, as well as a state-of-the-art industrial exhibition, bringing together nearly all robotic system vendors active at that time^4^. The success was further enhanced by opening the event to medical students to both encourage and survey the interest of the next generation in robotic surgery [3].

The Kurt-Semm-Center is dedicated to expanding the spectrum of robotic surgery, not only with regard to the portfolio, but also with respect to therobotic system armamentarium. Despite the clinical focus on da Vinci-Surgery, we are open to collaboration with different manufacturers of surgical systems. Clinical Studies with Dexter® and Symani® as well as preclinical studies conducted with Versius® and Senhance® are evidence of this.

## Numbers and figures

The available systems have been shared to allow maximum usage and output, which since 2013 has yielded over 8,000 robotic procedures in total. The yearly numbers in all robotic surgery programs have been rising consistently. The purchase of a third Xi system Dual Console in late 2024 has paved the way for a further increase in numbers and quality.

**Fig. 1:**
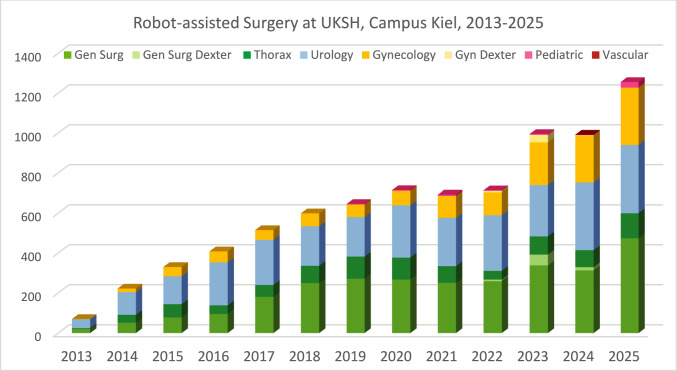
Quantitative development of robotic surgery at UKSH, Campus Kiel, 2013–2025

**Fig. 2:**
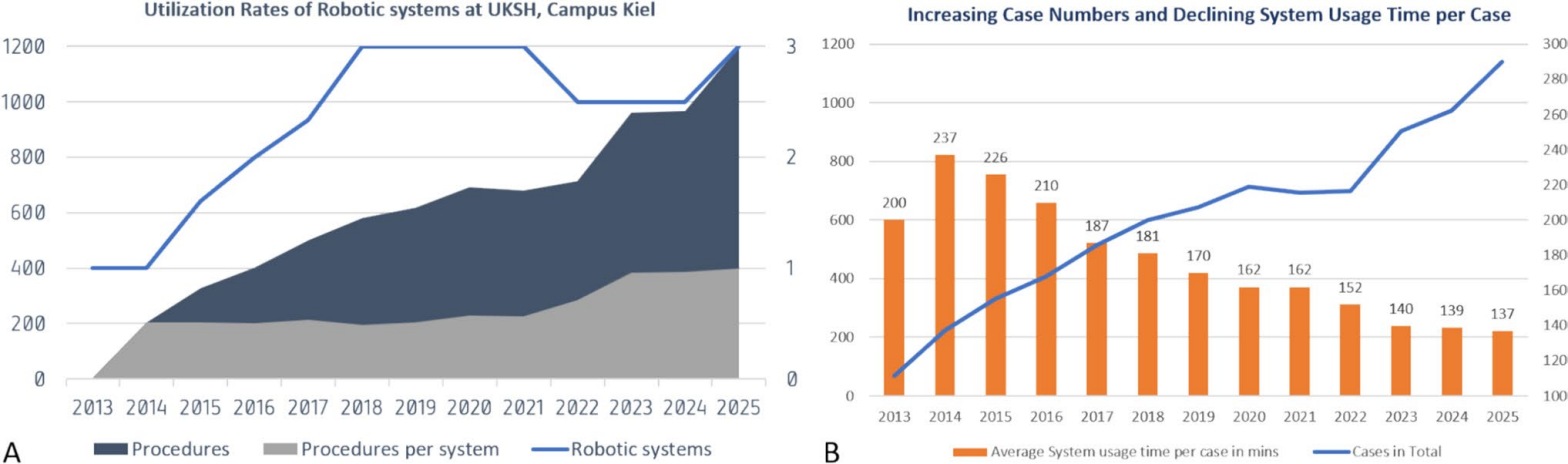
**A** Procedures, number of robotic systems and procedures per system; **B** Procedures and average system usage times per case

Overall, the numbers of robotic interventions have been rising consistently (Fig. 1). After a steep increase in the initial Si years, the yearly number reached a first peak in 2020 of nearly 700 procedures using 2 Si and 1 Xi platforms (2017–2022), followed by a small dent in 2021/22 due to the cancelation of elective interventions during the COVID-19 pandemic. Since then, the figures have peaked at nearly 1,000 in 2024 with two Xi-Systems (up to 90% system time utilization), and reached > 1,200 interventions in 2025 with 3 Xi-Systems (70–80% system time utilization). The average duration of system use per case has dropped from 3 h 46 min in 2015 to 2 h 17 min in 2025, thus a decrease by 39%—despite the extension of the portfolio to more complex interventions (Fig. 2).

**Fig. 3:**
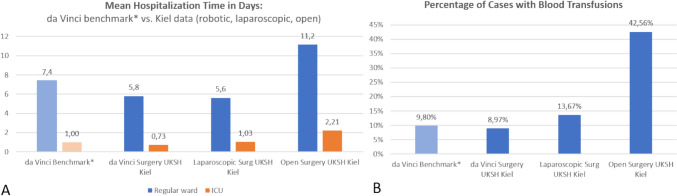
**A** 2023 hospitalization figures from a mix of predominantly complex interventions (da Vinci N = 702, Laparoscopic N = 214, Open 383, Source: § 21 KHEntgG, InEK). Da Vinci benchmark figures Germany. **B** Percentage of cases with blood transfusions in the same case portfolio

Moreover, figures from our hospital show below-average times of hospitalization and blood transfusion rates (Fig. 3).

## Clinical portfolio

### Robotic surgery and urology –an “old couple”

Robot-assisted surgery took off when urology took over the da Vinci robot – especially for robot-assisted prostatectomy which became an unchallenged surgical gold standard in the U.S. before 2010. The success story of robotic surgery, which was originally designed as a remote surgical solution to “stitch together” soldiers in distant battle fields, is thus partially owed to urology. The word was easily picked up by Klaus-Peter Jünemann, Head of the UKSH Urology Dept since 2001 and he did not rest until the hospital finally had two da Vinci Si systems in early 2013. Jünemann “fell in love” with robotic surgery and gradually changed the complete urological spectrum into a robotic routine, also developing many new techniques and specialties further, like, for example, transperitoneal partial nephrectomy [4], the robotic neobladder [5], the “Kiel duckbill” technique [6] for improved prolapse repair, extended salvage lymphadenectomy [7], and robotic vasovasostomy [8].

When P. Nuhn and his team followed Jünemann as Department leaders in 2023, they continued the tradition, while introducing their own focal points and techniques. The current team focuses on nerve-sparing prostatectomy, partial nephrectomy and robot-assisted cystectomy. They continue a more than 10-year experience of robotic cystectomies at the center, revealing the feasibility of this approach even in challenging, i. e. obese patient cohorts [9] Their special point of interest is the application of AI in various urological and surgical aspects [10–12]. A special AI workgroup has been founded to this end.

### Robot-assisted general surgery: From pioneer work to broad spectrum use in daily routines

The Department for General Surgery in Kiel was one of the first to develop and implement new specialties such as esophageal [13–15] and bariatric [16] surgery. The pioneer work, as well as collaboration with the Center for Clinical Anatomy, paid off [17, 18]. One of the milestones was the development of robotic esophagectomy. Under the guidance of Prof. Thomas Becker, hospital director and current Speaker of the Kurt-Semm-Center, Kiel has become one of the leading centers in Germany for robotic surgical treatment of obesity (> 900 cases), showing advantages in surgical time and fewer complications [19]. Moreover, Kiel has gathered outstanding expertise in robotic kidney transplants since 2015, with increasing numbers of live kidney donors. The aim is to establish a standardized pathway for robotic kidney transplant procedures involving both living and deceased kidney donors. Data are currently being collected and will be published in time. In addition, it is a high-volume center for combined pulmonological and thoracic surgery treatment of lung cancer. The cutting-edge Intuitive Ion® navigation tool is expected to become part of the robotic armamentarium soon. Similar applies to the fields of pancreatic, colorectal and hepatobiliary surgery, all of which have been developed and established as standardized robotic procedures. In 2018, we performed the first robotic pancreatic head resection in Germany [20] and demonstrated that robotic total mesorectal excision is superior to the laparoscopic approach [21, 22]. Robotic liver surgery was improved, e. g. by introducing ICG marking of the tumor [23]. The Department is dedicated to surgical training and proctoring and regularly welcomes guest surgeons from other clinics.

### Robot-assisted pediatric surgery–a new and growing field in the robotic spectrum

When asked why he chose Kiel to continue his surgical career, Chief Pediatric Surgeon R. Bergholz, stated that it was specifically because of the Kurt-Semm-Center and the opportunity to apply and develop robotic surgical routines for pediatric interventions. While da Vinci-assisted procedures have been successfully applied in children, the system faces certain limitations in pediatric use, as its standard 8 mm instruments and trocar sizes are not ideally suited for very small anatomical spaces. To address these challenges, the team has conducted research on the adaptability of various robotic systems for pediatric use: In collaboration with Asensus Surgical (formerly TransEnterix) and CMR Surgical pioneering experimental research with the Senhance® and Versius® robotic surgical systems was conducted. The pediatric surgeons established the first live piglet model <10 kg to assess the feasibility and safety of robotic platforms in neonatal and infant settings [24, 25]. The group is also the only one in Germany and among very few worldwide to establish a fetal sheep model for the development of prenatal treatment strategies for gastroschisis and congenital diaphragmatic hernia [26, 27]. In collaboration with researchers from Kiel University, novel minimally invasive instruments have been conceived, developed, and tested [28]. The pediatric surgery team also collaborates with the DFG-funded Research Center (SFB Biomagnetic Sensing) on the development of sensor-based innovations for more surgical precision and safety, resulting in two patent applications.

### Excellence in gynecological minimally invasive surgery

While gynecological surgery looks back at the longest tradition of minimally invasive surgery, it was also amongst the first disciplines to endorse robotic surgery. While robot-assisted hysterectomies have been the most common intervention at the beginning, the scope in the Kiel Department of Gynecology and Obstretics has been extended to include robotic standardized procedures for urogynecological prolapse repair (e. g. Burch colposuspension, sacrocolpopexy), endometriosis surgery, fertility surgery, neuropelveological surgery, radical gyne-oncology with lymphadenectomy, and myomectomy, while preserving equally refined skills and routines in conventional gynecological laparoscopy. The training in minimally invasive surgery, both robotic and laparoscopic, is a matter particularly close to the hearts of the gynecologists. Deputy Director Ibrahim Alkatout was the first in Germany to obtain a professorship for minimally invasive and robotic surgery. He is a dedicated and experienced da Vinci surgeon and proctor, and has also been appointed proctor by competing robotic system manufacturers like CMR surgical (Versius®). Since 2021 the Gynecology Department has been an official Intuitive Case Observation Center” and frequently hosts international surgeons for case observations. It also houses the Kiel School including a da Vinci SimNow® Training console. Apart from clinical research in surgical methodology, training research is one of the main focus points.

### Off-label robotic surgery for individualized patient care

Moreover, novel robotic disciplines such as vascular, maxillofacial and orthopedic spine surgery have performed robot-assisted procedures to provide individualized patient care. These interventions were carefully prepared in our regular robotic board meetings and were carried out with hands-on support from experienced robotic surgeons.

In 2019 da Vinci-assisted spine surgery was performed successfully by an interdisciplinary team [29]; in 2022 the world-first robotic parotid tumor extirpation took place [30]. A submandibular, minimally invasive access spared the young patient from having to undergo extensive cuts through his facial bones. In the field of vascular surgery, human and porcine models were used to investigate the extent to which the da Vinci system can be used to perform procedures on the coronary arteries and the thoracic aorta. Despite a lack of licensing from the system manufacturer, two da Vinci off-label aneurysm repairs of the aorta [31–34] were performed, as well as standard vascular reconstruction techniques with the Symani system.

### Clinical studies with other robotic surgery systems

Between 2022 and 2024, > 120 robot-assisted colorectal and gynecological surgical interventions were performed with the Dexter® System, which was developed by the Swiss Company Distalmotion as a hybrid surgical robot, aiming to unite the advantages of laparoscopic surgery with robotic precision and ergonomics [35–37]. With the advent of the Symani®, a novel surgical system emerged on the German market in the early 2020s. Due to its microsurgical approach in open surgical interventions and the advantages of miniaturized NanoWrist® instruments, it is an ideal tool in the hands of surgeons dealing with delicate structures in oral and maxillofacial surgery, ENT surgery, vascular surgery etc. It allows performance of ultrafine sutures in vessels, lymphatic vessels, and nerves down to 0.2 mm in diameter. Starting in 2022, more than 200 interventions have been performed in our hospital, most of them in the Department of Maxillofacial Surgery, which contributes to thePRIMO^5^ multi-center study [38]. Keen interest in the novel system has also been shown by the vascular surgeons [39, 40].

## Training

### Why training and training research are key issues

While surgical techniques and instrumentations have become more refined and, to a certain degree, more digitalized,—the most important skill, as well as the responsibility for the outcome, still lies with the human, i. e. with the surgeon controlling the instruments. This also applies if the control is exerted via a console of a robotic surgical system. Thus, training in surgical skills remains the key to safe surgical procedures that meet the demands of each individual case, regardless of current technological improvements that involve AI guidance or improved visualization of the surgical site.

### Kiel school

Training in minimally invasive surgery has a long tradition in Kiel, which goes back to Kurt Semm (cf. above). The “Kiel School of Gynecological Endoscopy” was founded in the 1990s, and has gained an international reputation under the guidance of Kurt Semm’s eleve Liselotte Mettler (b. 1939, recipient of the German Federal Cross of Merit 2025), followed by Thoralf Schollmeyer (b. 1962; d. 2014). Since 2014, Prof. Ibrahim Alkatout has been the head and driving force of the Kiel School. More than 200 international surgeons are trained in laparoscopic and endoscopic techniques every year. MIC basic and advanced courses comprise three days with a mixture of expert-taught seminars and surgical videos, surgical case observations and hands-on training on the hysteron/pelvitrainer with dry and wet lab models. The advanced anatomical classes, which also include intensive hands-on training on body donors at the Center of Clinical Anatomy of Kiel University, are a special highlight. As of 2025, classes in robotic gynecological surgery round off the training portfolio. All courses are certified by the German gynecological expert society AGE^6^.

### Center of clinical anatomy

The aforementioned Center of Clinical Anatomy of Kiel University has been a close partner of the Kurt-Semm-Center from the start. Apart from the above collaboration with the Kiel School, it offers numerous surgical trainings both in conventional laparoscopy and in specialized robotic da Vinci techniques, e. g. in esophageal, pancreatic and colon surgery [7]. Novel minimally invasive approaches have been developed, refined and integrated into surgical training workshops [41, 42].

### Kurt-Semm-Academy

The “Kurt-Semm-Academy” is a training center for robot-assisted surgery run chiefly in collaboration with Intuitive, which was founded under the former director Klaus-Peter Jünemann in early 2020 as a subdivision of the Kurt-Semm-Center. Despite the difficulties encountered during the COVID-19 pandemic, it never ceased being in operation. There have been over 700 trainings so far, with participants from Germany, Europe and overseas. While the minipig was the most common training model in the first five years, Kindheart®  animal organ systems, specifically prepared for this purpose, are now the dominant model. Nonetheless, Kurt-Semm-Academy offers a full surgical infrastructure set up in a former pediatric OR area, including two Xi-systems and anesthetic equipment. While the trainings so far are led by Intuitive, the next goal is to set up a curriculum for preclinical and clinical robotic training across the disciplines, with certificates issued by the Kurt-Semm-Center.

### Clinical training

Two of the current three da Vinci Xi-systems feature a dual console, which is invaluable for assisting and supervising young surgeons after formal training during their first robotic interventions or surgical steps. The recent implementation of Intuitive video recording (Intuitive Hub) and remote proctoring technology provides additional support to ensure thorough and continuous training as well as consistent patient safety.

### Training of surgical technologists/Assistive staff in the OR

The University Hospital in Kiel has a long tradition in the training of medical care staff and technical assistants in the UKSH Academy [8]. Robotic surgery has changed the demands and is now becoming a vital aspect of professional training. Robotic surgeons from the Kurt-Semm-Center support the training of this staff both in the class-room and in the OR. Moreover, we collaborate with Intuitive to provide more in-depth training for UKSH staff in the handling of surgical robots before, after and during surgery. The courses of the years 2019–2024 are summarized in the table below Table 1.

**Table 1:** Training figures of Kiel School, center of clinical anatomy and Kurt-Semm-Academy

Training in gynecological endoscopy (Kiel School)							
	**2019**	**2020**	**2021**	**2022**	**2023**	**2024**	**Total**
MIC Basic	37	47	55	75	106	118	**438**
MIC Advanced	40	14	39	41	31	27	**192**
**Training in robotic surgery in collaboration with Center for Clinical Anatomy (Kiel University)** in collaboration with DGAV*, Intuitive** et al							
Lap./Rob. Right Hemicolectomy* and Pancreatoduodenectomy **^/^*				6	29	12	**47**
Robotic Esophagectomy (RAMIE)*	7			9	8		**24**
**Training in robotic surgery (Kurt-Semm-Academy, collaboration with Intuitive)**							
Robotic surgery training for doctors		27	115	164	172	185	**663**
Robotic surgery training for OR staff						25	**48**

### Training research

Not only the training itself, but also training research remains a key issue, which has been consistently examined in various studies driven by the Kiel School. Most recently, a study examined the tendency of senior surgeons to defend their “own” methods against new findings during proctoring sessions [43]. A SimNow stand-alone simulation console was let by Intuitive for training and research purposes and is now permanent equipment; it was updated in 2025 with the latest generation of highly realistic simulation software. Moreover, a project with the Institute for Medicinal Psychology has led to studies regarding the aptitude of medical students and interns for surgical disciplines [44], similarly with the Center of Clinical Anatomy [45]. Current studies involve the Institute of Medical Ethics and the Fraunhofer IMTE Lübeck.—We firmly believe that training in robotic surgery has to keep pace with the fast development and roll-out of this surgical technique which is now becoming a surgical standard in nearly all hospitals. This also means that training needs to be tailored to each robotic surgical system and encompass the technological progress each new system generation. But beyond this, the target group of training must be extended; the focus should include not only the “new” or “young” robotic surgeon, but also the older non-robotic surgeon and even the experienced robotic surgeon. Finally, robotic surgery must become an integral part of the medical curriculum, with an early identification of the surgical talents.

## Funded research

The clinical and preclinical research output comprises more than 200 peer-reviewed publications, many of which were based on in-house projects and single or multi-center studies with a reference to robotic and minimally invasive surgery. In addition to the two strongholds of inner-clinical collaboration and training, experimental research based on an increasingly tight-knit collaboration with the Technical Department of Kiel University became a third column of the Kurt-Semm-Center. The potential yields of collaborating in the fields of augmented Reality, artificial intelligence, imaging, navigation, sensorics and material sciences became increasingly obvious, and the chance to use this to the best advantage came when a funding opportunity from residual REACT-EU funds (EFRE) came into sight. A total of €3.4 million was granted for a project and research platform named “OP der Zukunft” (OR of the Future) under the leadership of Klaus-Peter Jünemann. The consortium consisted of the University Hospital Schleswig–Holstein (Kurt-Semm-Center and Clinic for Nuclear Medicine), the Technical Faculty of Kiel University (ET&IT, Computer Science, and Material Science), and the industrial partners Vater Solution GmbH and MiE (Medical Imaging Electronics). The ambitious goal of the first pilot was to register the CT image with the endoscopic image. Custom-made phantoms with PET-CT-properties were conceived to this end, a patent application was filed [46]. In addition, various tracking methods were combined in an experimental lab setup: a ceiling-mounted optical tracking system, electromagnetic tracking, 3D scanners and 3D measuring equipment. The platform is rounded off by a robotic surgical system (Dexter), videoendoscopy and a Lambda® robotic steering console enabling haptic feedback. We also explored the possibility for a primary and secondary robot to interact in the same work sphere [47, 48] to discuss the possibility of a second robot in the OR, to be used, for example by the surgical assistant.

The follow-up project “TWIN-WIN” echoes the use of digital twins. A CT image can be seen as a digital twin of the patient’s body and his/her organs inside. The aim was to take image registration further to enable not only static objects to be matched, but to morph the CT image to the real-time deformed constellation of the organs during the surgical process. Predictive AI, ultrasound imaging in the OR, and sensor fusion algorithms were seen as possible keys to solving the problem. A sub-objective was to optimize the endoscopic image, e. g. through smoke removal [49]. Predefined landmarks have to be identified manually or eventually by AI to get the best possible match, which may have the potential to optimize surgical navigation in soft tissue surgery in the future. The consortium was extended to include specialists from Lübeck University’s Institute for Robotics and Cognitive Systems. The project is funded by the state of Schleswig–Holstein with nearly one million Euros (2024–26).

## Outlook

Robotic surgery has a history of some 25 years of successful clinical implementation. The roll-out in the United States, in Europe and many other parts of the world has been massive. However, it is still far from stagnation, as more and more entities and surgical disciplines become robotic. As it becomes more prevalent and more competitive, it also becomes cheaper,—or at least less expensive. The new generation of robotic systems increasingly includes AI features like expert systems, improved navigation systems, safety channels, or haptic feedback; the latter and some of the former have already been implemented in the da Vinci 5-system generation. Other systems and niche solutions are competing against, respectively supplementing the current surgical possibilities.

The most radical change to be expected is that surgery will become digitalized and robotized at large, displacing traditional open surgery and even laparoscopy. Even emergency interventions can now often be done robotically. The next steps are the robotization of smaller and non-complex interventions,—where usage of the robot is possible, but currently restricted by economic considerations,—and finally, of outpatient surgery. While the financial coverage of the latter remains doubtful, our goal is to keep pace with new developments in robotic surgery. Not only by maintaining state-of-the-art procedures, but also by developing new techniques and methods, both in the OR and in surgical training. The above-mentioned projects have helped to initiate close collaboration with the technical disciplines at Kiel University. Prior research results can be used to implement virtual training solutions and to develop customized phantoms for experimental surgery, training, and surgical planning.

The University Hospital of Schleswig–Holstein features the asset of having two Campuses in the two largest cities of the region, Kiel and Lübeck. On both campuses, the impact of robotic surgery has become visible and tangible in the form of innovation platforms with daily collaborative research involving both clinical and technical experts. In Lübeck, the surgical innovation platform LIROS has been shaped through the close collaboration between the robotic surgeons and Fraunhofer Institute IMTE, flanked by Fraunhofer MEVIS and DFKI. Cross-campus collaboration between the hubs that are dedicated to different aspects of robotic surgery is creating new impetus for small and large-scale projects. Structures for a common ground between the chief players on both campuses have been laid, ready for use. The next logical project is to develop and test technical concepts for remote surgery from one campus to another, with the prospect of enabling expert surgical care even in remote rural areas.

## Conclusion

The Kurt-Semm-Center has always followed the credo of establishing the best surgical technology for the best possible patient treatment. Being a university hospital, surgical care is always seen in close connection with medical/surgical training;—and the ambitious claim to offer not only state-of-the-art surgery, but also to advance it further through studies and research, both experimental and clinical.

We are open to technological innovation, without bias or self-interest, and we are interested and willing to consider and test innovative surgical technologies regardless of the producer, always with the goal to identify the best technology from the patients’ perspective. Sometimes the best system for one patient group is not the best for another.

The road was often paved with obstacles, i. e. bureaucratic traps, lack of funding, personal vanities, doubts, etc. Setting up a training platform or getting the funding and support for yet another robotic system was in every single case a process of years rather than months, and often felt like a mission impossible. However, the key to success has been co-working across the disciplines, with continuing tenacity and dedication to state-of-the-art minimally invasive surgery. We believe that many hospitals could profit from an interdisciplinary spirit, having realized that individual department-specific interests are eventually met better when people learn from each other and work as a team.

## Data Availability

All data supporting the findings of this study are available within the paper or in the References section. We have used the platform “My Intuitive” for compiling and analysing our da Vinci case numbers and system usage.

## Abbreviations

UKSH: Universitätsklinikum Schleswig–Holstein (University Hospital Schleswig-Holstein)
AGE: Arbeitsgemeinschaft Gynäkologische Endoskopie (Society for Gynecological Endoscopy)
LIROS: Lübeck Innovation Hub for Robotic Surgery
IMTE: Institut für individualisierte und zellbasierte Medizintechnik (Fraunhofer research institution for individualized and cell-based medical engineering)
MEVIS: Medizinische Diagnosesysteme und Visualisierung (Fraunhofer institute for digital medicine)
DFKI: Deutsches Forschungszentrum für KI (German research center for artificial intelligence)

## Footnotes

1 We owe gratitude to UKSH administration and the Medical Faculty of Kiel University for supporting this project and helping us to finance the initial years as well as the robotic equipment.

2 Prof. Dr. Klaus-Peter Jünemann, b. 29 Sept 1956 in Witzenhausen (Hessia); d. 29 Aug 1923 in Kiel, Academic/Medical Career at the University of Heidelberg, 2001-2022 Director of the Dept. of Urology and Pediatric Urology at the University Hospital Schleswig-Holstein, Campus Kiel.

3 Prof. Dr. Kurt Semm, b. 23 March 1927 in Munich, d. 16 July 2003 in Tucson, Arizona, 1970-95 Director of the Dept. of Gynecology in Kiel University Hospital.

4 Intuitive, CMR Surgical, Avatera.

5 https://www.mmimicro.com/portfolio/primo-clinical-study.

6 https://www.uksh.de/kiel-school/Kurse.html.
